# Inhibiting stemness and invasive properties of glioblastoma tumorsphere by combined treatment with temozolomide and a newly designed biguanide (HL156A)

**DOI:** 10.18632/oncotarget.11595

**Published:** 2016-08-25

**Authors:** Junjeong Choi, Ji-Hyun Lee, Ilkyoo Koh, Jin-Kyoung Shim, Junseong Park, Jeong Yong Jeon, Mijin Yun, Se Hoon Kim, Jong In Yook, Eui Hyun Kim, Jong Hee Chang, Sun Ho Kim, Yong Min Huh, Su Jae Lee, Michael Pollak, Pilnam Kim, Seok-Gu Kang, Jae-Ho Cheong

**Affiliations:** ^1^ Department of Pharmacy, College of Pharmacy, Yonsei Institute of Pharmaceutical Sciences, Yonsei University, Incheon, Republic of Korea; ^2^ Brain Korea 21 Plus Project for Medical Science, Yonsei University, Seoul, Republic of Korea; ^3^ Departments of Neurosurgery, Brain Tumor Center, Severance Hospital, Yonsei University College of Medicine, Seoul, Republic of Korea; ^4^ Department of Bio and Brain Engineering, Korea Advanced Institute of Science and Technology, Daejeon, Republic of Korea; ^5^ Departments of Nuclear Medicine, Severance Hospital, Yonsei University College of Medicine, Seoul, Republic of Korea; ^6^ Departments of Pathology, Severance Hospital, Yonsei University College of Medicine, Seoul, Republic of Korea; ^7^ Department of Oral Pathology, Yonsei University College of Dentistry, Seoul, Republic of Korea; ^8^ Departments of Radiology, Severance Hospital, Yonsei University College of Medicine, Seoul, Republic of Korea; ^9^ Department of Life Science, Research Institute for Natural Sciences, Hanyang University, Seoul, Republic of Korea; ^10^ Department of Oncology and Medicine, McGill University, Gerald Bronfman Centre, Montreal, Quebec, Canada; ^11^ Department of Surgery, Severance Hospital, Yonsei University College of Medicine, Seoul, Republic of Korea

**Keywords:** biguanide, glioblastoma, HL156A, invasion, tumorsphere

## Abstract

Studies have investigated biguanide-derived agents for the treatment of cancers and have reported their effects against tumorspheres (TSs). The purpose of this study was determining the effects of HL156A, a newly designed biguanide with improved pharmacokinetics, on glioblastoma TSs (GMB TSs) and assess the feasibility of this drug as a new line of therapy against glioblastoma, alone or combined with a conventional therapeutic agent, temozolomide(TMZ). The effects of HL156A, alone and combined with TMZ, on the stemness and invasive properties of GBM TSs and survival of orthotopic xenograft animals were assessed. HL156A, combined with TMZ, inhibited the stemness of GBM TSs, proven by neurosphere formation assay and marker expression. Three-dimensional collagen matrix invasion assays provided evidence that combined treatment inhibited invasive properties, compared with control and TMZ-alone treatment groups. TMZ alone and combined treatment repressed the expression of epithelial-mesenchymal transition-related genes. A gene ontology comparison of TMZ and combination-treatment groups revealed altered expression of genes encoding proteins involved in cellular adhesion and migration. Combined treatment with HL156A and TMZ showed survival benefits in an orthotopic xenograft mouse model. The inhibitory effect of combination treatment on the stemness and invasive properties of GBM TSs suggest the potential usage of this regimen as a novel strategy for the treatment of GBM.

## INTRODUCTION

Glioblastoma (GBM) is the most aggressive malignant brain tumor, with patients showing a very limited survival rate despite the best treatment [1]. According to National Comprehensive Cancer Network (NCCN) guidelines on central nervous system cancer, only a third of GBM patients survive for 1 year and less than 5% live beyond 5 years [2]. Temozolomide (TMZ), an alkylating (methylating) agent, is now the standard of care in conjunction with postoperative radiation therapy for younger, good-responding patients with GBM [1, 2]. However, the disease is invariably fatal, and patients ultimately succumb because of disease relapse. A growing body of evidence supports the idea that cancers are initiated and maintained by a subpopulation of cells termed cancer stem cells (CSCs) [3, 4]. The clinical implication of research on tumor-derived, CSC-enriched tumorspheres (TSs) is that a curative therapy will require complete elimination of this unique population, even in patients with an initial positive response to treatment, since the disease may ultimately recur if even a small number of CSCs survive the therapy [5, 6]. Accumulating evidence has established that CSC populations are more resistance to conventional cancer therapy than non-CSC populations [7]. For example, CD133-positive GBM TSs display a strong capacity to resist chemotherapy and radiotherapy [8, 9]. Consequently, novel therapeutic strategies designed to eliminate or inhibit CSCs, including targeting surface markers or signaling cascades, or altering the microenvironment in which these cells potentially reside, have been explored [10].

Studies on the biguanide derivate, metformin (N′, N′-dimethylbiguanide), the most widely used oral therapeutic agent for lowering blood glucose concentration in patients with type 2 diabetes, have revealed that this agent significantly reduces cancer incidence and improves cancer patients' survival. Laboratory evidence of the antineoplastic effects of biguanide has continued to accumulate, and results of the first generation of clinical trials on metformin are anticipated [11, 12]. A number of mechanisms have been suggested to account for the direct actions of biguanides on transformed cells or cells at risk for transformation, including induction of energetic stress and consequent disruption of cellular homeostasis, energy depletion caused by inhibition of oxidative phosphorylation (OXPHOS), which leads to an energy-conserved state, and activation of intracellular 5′ AMP-activated protein kinase (AMPK), but the precise mechanism is still under investigation. Remarkably, Hirsch et al. have demonstrated that mass-forming, self-renewing, tumor-initiating breast cancer cells exhibit an exaggerated sensitivity to metformin [13]. This group suggested that transforming growth factor (TGF)-β–induced epithelial-mesenchymal transition (EMT) might represent a common molecular mechanism underlying the anti-CSC actions of metformin [11]. However, supporting evidence for this concept is still limited. Metformin has limitations as an anticancer drug because its hydrophilic nature prevents it from entering cells [14]. In addition, metformin only enters cells through members of the organic cation transporter (OCT) family, whose expression is dependent on the genetic background of individuals [14].

HL156A is a derivative of metformin with increased hydrophobicity developed by HanAll Biopharma in Korea., but its potency and pharmacokinetic property were improved [15]. The compounds were evaluated the potency of inhibiting OXPHOS in mitochondria and pharmacokinetic property *in vivo* [15]. The toxicity of HL156A was assessed and there were no meaningful toxicities with up to a 120 mg/kg dose [15]. It was reported that HL156A is protective against peritoneal fibrosis in an *in vitro* and *in vivo* model of peritoneal fibrosis [15].

In the present study, we assessed the effects of the newly designed biguanide, HL156A, alone and combined with the conventional chemotherapeutic agent TMZ, on the tumorsphere properties and survival of orthotopic xenografted animals. Our results suggest the feasibility of using HL156A as part of a new therapeutic regimen for GBM.

## RESULTS

### GBM TSs characterization

**Figure 1:**
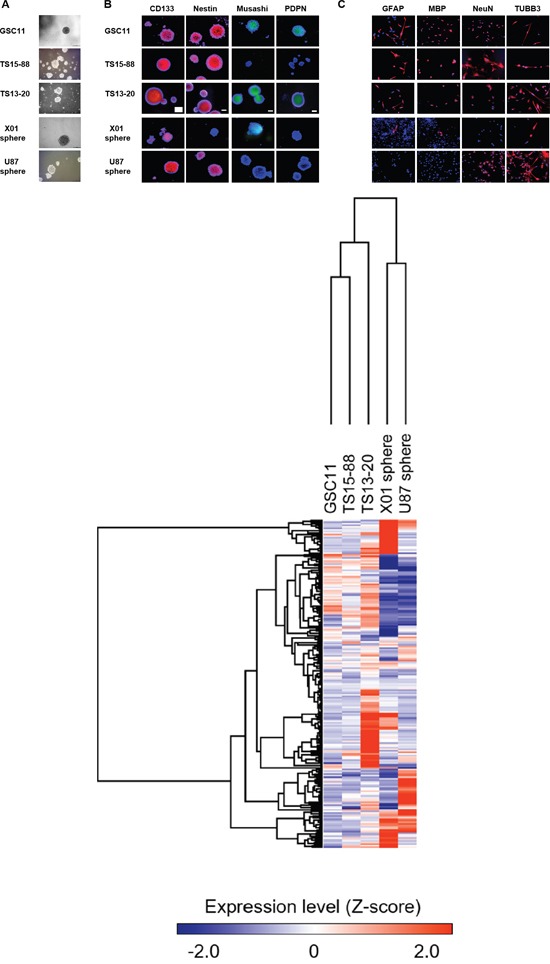
Glioblastoma tumorspheres with marker expression of stemness and neuroglial differentiation **A.** GSC11, TS15-88 and TS13-20 GBM-TSs, and sphere-cultured U87 and X01 cells. **B.** Expression of stemness markers in GBM-TSs. All GBM TSs express all or some stemness markers. **C.** Neuroglial differentiation of GBM-TSs. **D.** Unsupervised clustering of gene expression of GBM-TSs.

The self-renewal capacity was confirmed in GSC11, TS15-88, TS13-20, U87 sphere, and X01 sphere igure 1A). Immunocytochemistry revealed that all four cell tyspes were positive for CD133 (also known as PROM1), and GSC11, TS15-88 and U87 spheres were also positive for nestin. Musashi expression was variable, with only GSC11 and X01 TSs showing partial expression. Podoplanin (PDPN) expression was observed only in GSC11 TSs (Figure 1B). Neuroglial cells were identified in all GBM-TSs, as evidenced by the expression of glial fibrillary acidic protein (GFAP), major basic protein (MBP), NeuN and/or TUBB3 (Figure 1C). GFAP and MBP expression was not identified in U87 sphere. GFAP and MBP expression were not detected in sphere-cultured U87 cells. The molecular characteristics of GBM-TSs, including molecular subtype based on expression profiles, methylation of the O(6)-methylguanine-DNA methyltransferase (MGMT) gene promoter and the presence of IDH mutations, are summarized in Table 1. An analysis of the gene expression profile of these GBM-TSs by microarrays and unsupervised clustering revealed that X01 and U87 spheres showed an expression profile distinct from that of GSC11 and TS15-88 TSs, as well as TS13-20 TSs (Figure 1D).

**Table 1:** Molecular characteristics of glioblastoma tumor spheres

Cell	Molecular Subtype	Molecular Marker	
		IDH1	MGMT
GSC11	Classical	WT	Unmethylation
TS15-88	Classical	WT	Unmethylation
X01	Mesenchymal	N/A^*^	N/A^*^
TS13-20	Neural	WT	Methylation
U87 sphere	Mesenchymal	WT	Methylation

N/A

* Assay failed

### Chemical structure of HL156A and *in vivo* blood-brain barrier permeability

**Figure 2:**
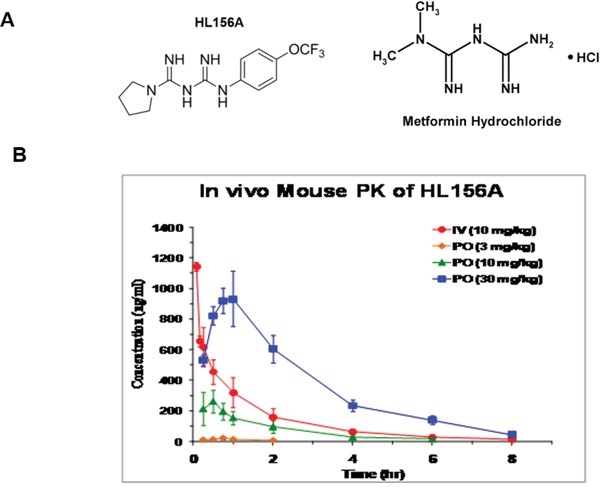
The chemical structure and pharmacokinetics of HL156A **A.** The structures of HL156A and metformin. HL156A contains the central biguanide moiety of metformin. **B.** Pharmacokinetics of HL156A. The bioavailability of the drug was determined to be ~72.9% following administration of a dose of 30 mg/kg in mice.

The chemical structure of HL156A (N-[N-{4-(trifluoromethoxy) phenyl} carbamimidoyl] pyrrolidine-1-carboximidamide acetate), a derivative of biguanide with improved bioavailability, is depicted in Figure 2A. In contrast to metformin with limited brain-blood barrier permeability due to the heterogenous expression between the individuals, HL156A enters the cell independent of OCT1 and penetrates extensively into the brain. HL156A is cell membrane permeable and orally available *in vivo*. Following oral administration (30 mg/kg) to test mice, the brain-to-plasma (BP) ratio of HL156 (0.37) was higher than that of metformin (0.10) and phenformin (0.13), and its bioavailability was ~72.9%, revealing improved penetration into the central nervous system (Figure 2B, Table 2 and Supplementary Table 1).

**Table 2:** Blood brain barrier permeability of HL156A

Compound	Time	Plasma(ng/mL)	Brain(ng/g)	BP ratio (Cbrain/Cplasma)
HL156A	0.5	582.67	99.2	0.17
	3	172.33	63.07	0.37
Metformin	0.5	583.5	74	0.13
	3	534.33	50.13	0.1

### Selection of HL156A and TMZ concentrations for experiments

**Figure 3:**
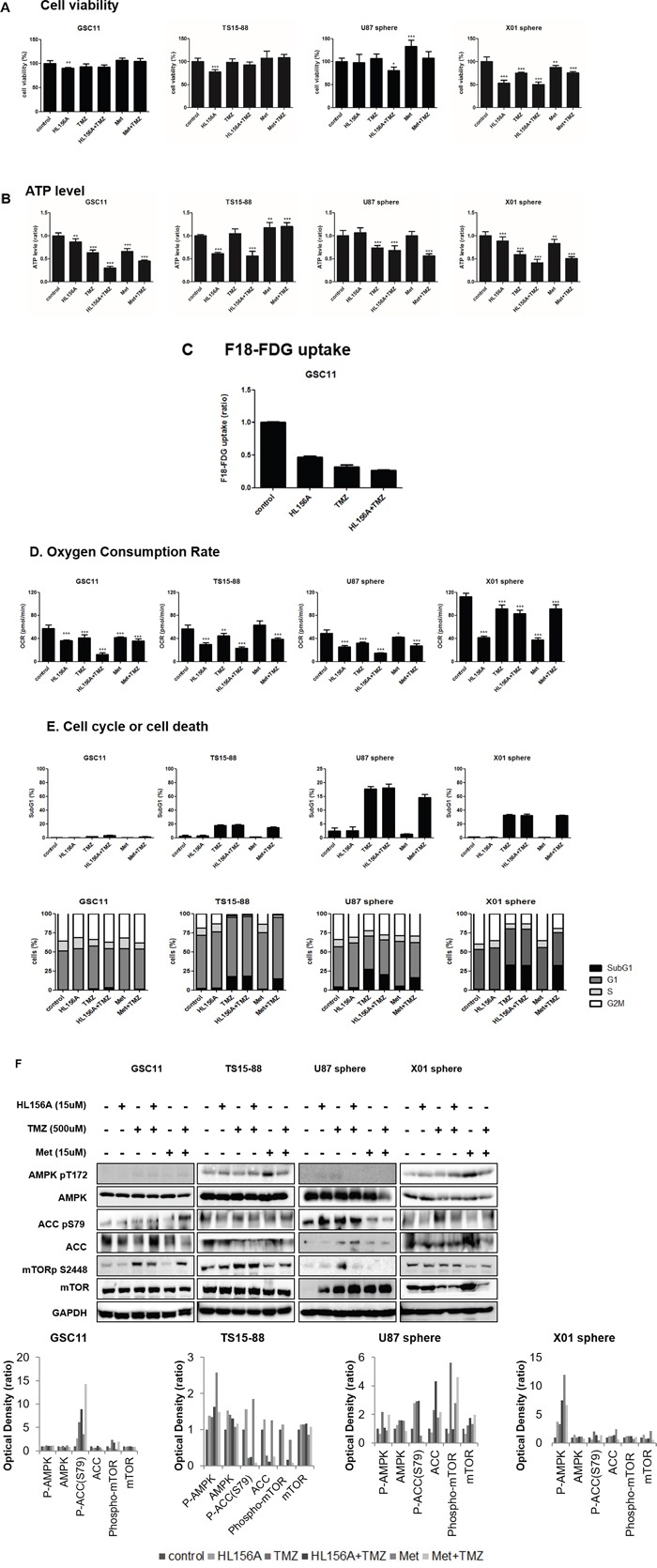
Effects of HL156A, TMZ, and combination treatment on cell viability **A.** HL156A and TMZ alone, at concentrations of 15 and 500uM, respectively, showed minimal effects on cell viability, enabling us to assess the cellular mechanism responsible for the effects of HL156A. **B.** ATP level was decreased on the treatment of HL156A, TMZ and HL156A+TMZ. **C.** ^18^F-FDG uptake was markedly decreased in GBM-TSs treated with HL156A, TMZ, or both. The decrease in FDG uptake was most prominent in the combination-treatment group, suggestive of a low metabolic status. **D.** Oxygen consumption rate was markedly decreased by treatment with HL156A or TMZ, and was prominently observed in the combination treatment group. Similar findings were observed with metformin plus TMZ treatment. **E.** Treatment with HL156A or HL156A+TMZ did not cause cell-cycle arrest or definite apoptotic cell death at the tested concentration. **F.** Effects of HL156A, TMZ, and combination treatment on AMPK and the mTOR pathway. Although biguanide is known to act as an AMPK agonist and inhibitor of mTOR signaling, neither AMPK activation nor subsequent mTOR inhibition was consistently observed, suggesting that AMPK-mTOR is not the major pathway by which HL156A and HL156A+TMZ act.

To identify particular drug-induced cellular phenomena without affecting the viability of cells, we established a sublethal concentration of each drug. To this end, we examined cell viability in GBM-TSs (GSC11, TS15-88 and X01) and sphere-cultured U87 GBM cells following treatment with HL156A and TMZ, alone and in combination, using a 3-(4,5-dimethylthiazol-2-yl)-5- (3-carboxymethoxyphenyl)-2-(4-sulfophenyl)-2H-tetrazolium (MTS) assay. HL156A and TMZ at concentrations of 15 and 500 μM, respectively, which showed minimal effects on cell viability (>80%) in three out of the four cell types, were adopted as sublethal concentrations for subsequent experiments (Figure 3A). The combination treatment was additive, but not synergistic.

### Effects of HL156A, TMZ, and combination treatment on cellular metabolism of GBM TSs and AMPK-mTOR pathway

Given that HL156A was originally developed as an AMPK agonist, we assessed the effects of HL156A, TMZ and their combination on cellular metabolism by assaying ATP, 18F-FDG uptake and oxygen consumption rate (OCR) (Figure 3B, 3C, and 3D). Even at a concentration that does not affect viability, treatment with HL156A, TMZ or their combination decreased ATP levels, 18F-FDG uptake and OCR, suggestive of metabolic perturbations in cells and energy stress. The decrease was most prominent in the combination treatment group. At the concentration examined, TMZ alone and in combination with TMZ caused a minimal increase in apoptosis, but had no clear G2/M-blocking effect; this contrasts with a previous report showing that an AMPK agonist blocks G2/M in cells (Figure 3E) [16] An examination of GBM-TSs revealed inconsistent expression of AMPK-mTOR pathway proteins. AMPK phosphorylation and subsequent mTOR inhibition in response to the drugs was not observed in tested GBM-TSs, despite ATP depletion in these cells (Figure 3F). There was no previously known LKB1 gene mutation in GSC11 cell line when we analyzed exon 1,2,3,5 and 7, except one deletion on exon 3 which is not described in other disease.

### Effects of HL156A, TMZ, and combination treatment on the stemness of GBM TSs

**Figure 4:**
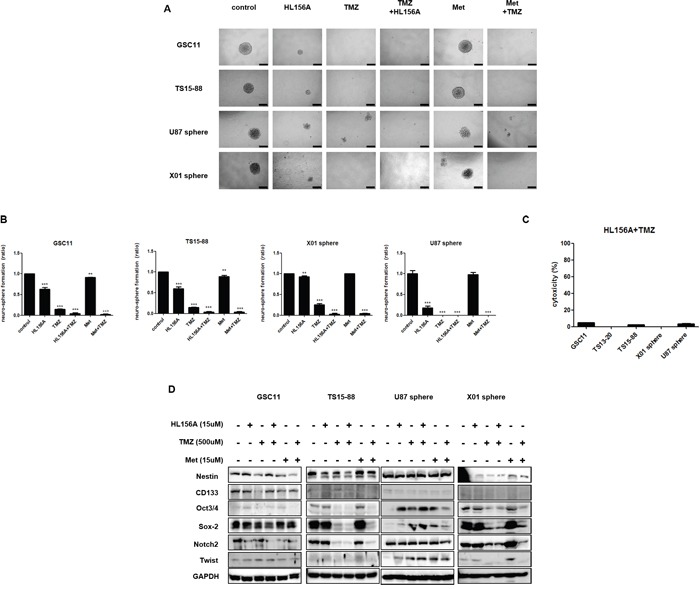
Effects of HL156A, TMZ, and combination treatment on stemness and neuroglial differentiation of GBM-TSs **A, B.** Effects of HL156A and combination treatment with HL156A and TMZ on stemness were assessed using tumorsphere-formation assays (A) and quantified (B), revealing a decrease in the number of neurospheres. **C.** Cytotoxicity during tumorsphere-formation assays was minimal, as evidenced by the results of LDH assays. **D.** Combination treatment with HL156A and TMZ decreased expression of the stemness markers Oct3/4, Sox2, and Notch2.

HL156, TMZ and combination treatment decreased the stemness of GSC11, TS15-88, U87 and X01 TSs, as demonstrated by tumorsphere-formation assays (Figure 4A and 4B) and Western blotting for stemness markers, including nestin, CD133, Sox-2, Oct3/4, Notch 2, and Twist. The proportion of sphere-positive wells was markedly decreased by treatment with HL156A or TMZ alone, and was more prominently decreased by combined treatment with HL156A and TMZ (Figure 4B). This latter effect was additive, although there was a trend towards synergy that fell short of significance. LDH assays revealed that a majority of sphere cells were viable, implying that the decrease in sphere formation is not attributable to cell death (Figure 4C). Combined treatment with Hl156A and TMZ caused a decrease in Sox2 and Notch2 expression in GSC11 TSs, and decreased Oct 3/4, Sox2, Notch 2 expression in TS15-88 and X01 TSs (Figure 4D). This effect was not attributable to changes in cellular viability or cytotoxic effects of drugs, as evidenced by the fact that LDH assays also revealed no significant cell death in HL156A, TMZ or combined treatment groups (Figure 4C). Neither HL156A nor TMZ promoted neuro-glial differentiation, as evidenced by the absence of a change in the expression of the neuronal markers Olig2, Tuj1, and GFAP (Supplementary Figure 1). Thus, it is unlikely that the therapeutic effect of these agents is mediated by differentiation of tumor cells into mature cells—one proposed mechanism for targeting CSCs.

### Effects of HL156A, TMZ, and combination treatment on the invasive properties of GBM TSs and marker expression of epithelial mesenchymal transition

**Figure 5:**
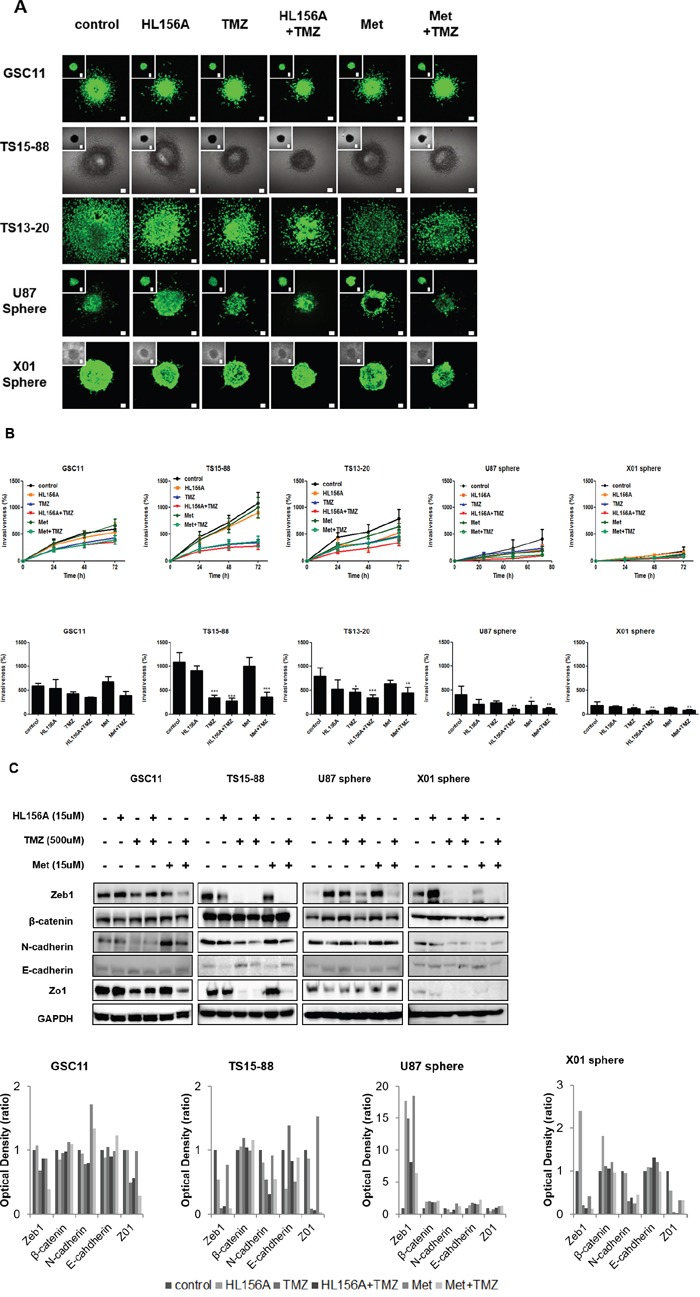
Effects of HL156A, TMZ, and combination treatment on the invasive properties of GSC11, TS15-88 and TS13-20 GBM-TSs, and sphere-cultured U87 and X01 cells **A, B.** 3D collagen matrix invasion assays show decreased invasiveness of cells implanted together with HL156A or HL156A + TMZ (A), as quantified in B. **C.** Expression of EMT pathway-related markers was altered by treatment with HL156A, TMZ or both, an effect that was most prominent with combination treatment.

We evaluated the effect of drugs on the invasive property of GBM TSs using three dimensional (3D) matrix platforms and quantified the result by assessing the area of invasion of GBM TSs. The implanted GBM TS migrated radially into the collagen matrix, relevant to *in vivo* tumor behaviors. The treatment with HL156A, TMZ or a combination of both drugs suppressed the invasiveness of GSC11 GBM TSs compared with controls (Supplementary Videos S1 and S2), an effect that was most prominent with combination treatment, when assessed quantitatively (Figure 5A and 5B, implanted cells and drugs together) This invasion-suppressing action was not attributable to drug effects on cell viability since cell viability was not significantly affected under these experimental conditions. To identify mechanisms underlying of inhibition of invasion, we assessed expression of the EMT-related markers, β-catenin, Zeb1 and N-cadherin, and the mesenchymal-epithelial markers E-cadherin and Zo-1. These markers showed inconsistent expression among cell types, with TMZ and combination treatment causing decreased N-cadherin expression in GSC11 cells, and decreased Zeb1 and N-cadherin expression in TS15-88 and X01 cells; the latter observation may provide a mechanism to account for the results of 3D invasion assays described above (Figure 5C).

### Gene expression microarray and gene ontology analysis

**Figure 6:**
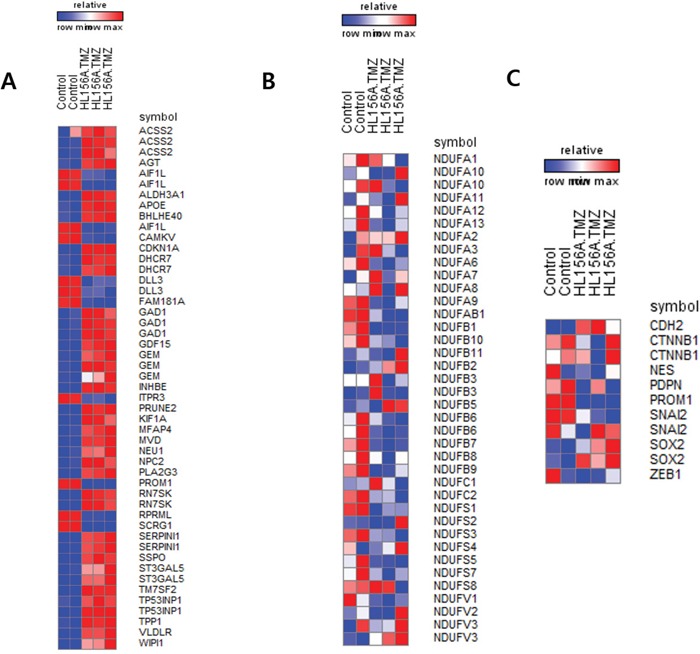
High-throughput gene expression microarray **A.** Differentially expressed genes between control and the combination treatment group. **B.** Expression of mitochondria complex I genes. Some genes were down-regulated in the combination treatment group. **C.** Expression of selected genes. CD133 (PROM1), podoplanin (PDPN), and ZEB1 were down-regulated in the combination treatment group, consistent with the results of Western blot analyses.

Transcriptome analyses was performed using Illumina HumanHT-12 v4 Expression BeadChips, and a heatmap of top-ranked differentially expressed genes was generated (Figure 6). These analyses identified genes that were differentially expressed following combination treatment. Among several downregulated genes were those encoding FBLN7, an adhesion protein that interacts with extracellular matrix, Lyn, a protein known to regulate cell migration, and LAMA4, a type of laminin. Using a BRB-Array tool to perform a Gene Ontology analysis of genes that were differentially expressed between TMZ alone and combined treatment with HL156A and TMZ, we found that the differentially expressed gene set included genes related to cell adhesion, cell migration, cell motion, and regulation of cell adhesion (Supplementary Table 2). Consistent with previous reports that biguanide blocks mitochondrial complex I, we found that combined treatment with HL156A and TMZ down-regulated several genes belonging to mitochondrial complex I. We also observed that stemness markers (CD133/PROM1, ZEB1 and PDPN) were down-regulated whereas E-cadherin (CDH2) expression was up-regulated in the combination treatment group.

### Effects of HL156A on xenograft tumor growth

**Figure 7:**
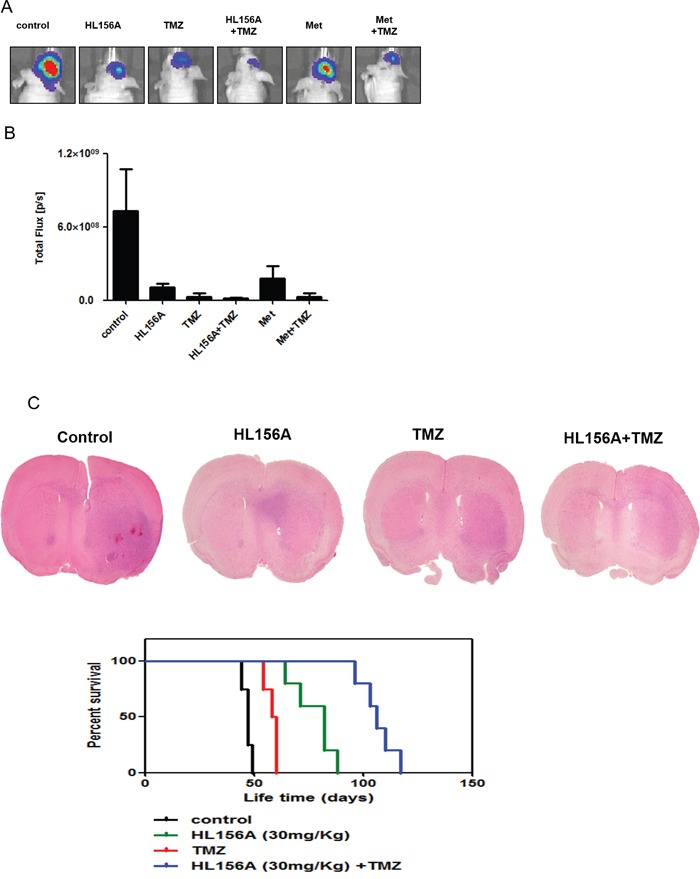
Effects of HL156A and combined HL156A and TMZ treatment on xenograft tumor growth and animal survival **A, B.** GSC11-luc TSs were xenografted and tumors from animals treated with HL156A (35 mg/kg) or TMZ (30 mg/kg) alone, or HL156A and TMZ (45 and 30 mg/kg, respectively) in combination were assessed using bioluminescence imaging (n=5 for each group) Tumor volumes were assessed and quantified. **C.** Tumors from animals treated with HL156A or HL156A and TMZ showed less irregular margins than those from controls. Notably, combination treatment decreased tumor volume. Survival of animals in the combination treatment group was significantly increased (P < 0.0001).

Finally, we analyzed the survival of orthotopic xenograft mice. Median survival times of controls and mice treated with HL156A, TMZ and HL156A+TMZ (n=5 per each group) were 47, 82, 58 and 106 days, respectively. Separately, bioluminescence images of mice treated with drugs were analyzed and quantified (Figure 7A and 7B). After sacrificing animals, brains were removed, sectioned, and stained with H&E. Although combination treatment did not prevent the formation of tumors, it limited the size and extent of tumor masses (Figure 7C). As shown in Figure 7C, these *in vivo* experiments revealed that combined treatment with HL156A and TMZ exerted a significant beneficial effect on the overall survival of animals (P < 0.001, log rank test). The survival benefit was reproduced at a higher dose of HL156A (45 mg/kg, P < 0.001; Supplementary Figure 2).

## DISCUSSION

Despite recent progress in our understanding of GBM, the prognosis for GBM patients remains grim [17]. Even in cases where surgery appears to achieve complete resection, the tumor tends to recur and ultimately takes the patient's life [18–20]. Accordingly, a new therapeutic strategy would seem to be required. Identifying key molecules associated with the unique properties of tumor cells so as to target these cells may be a viable approach, since numerous studies have reported that tumorspheres derived from GBM are chemo- and radio-resistant, which could potentially cause recurrence of the disease.

In the present study, we examined the effects of treatment with HL156A and TMZ, alone and in combination, on the stemness and invasive properties of GBM TSs (GSC11, TS15-88, TS13-20, U87 sphere and X01). Combined treatment with the new agent HL156A and the well-known conventional chemotherapeutic agent TMZ reduced stemness and invasive properties of GBM-TSs, an effect that was associated with changes in EMT-related markers. A similar additive effect of metformin and conventional therapy has been reported for a breast cancer stem cell model [21] and glioblastoma model [22, 23]. The authors of former study reported an additive effect of metformin with the cytotoxic effects of hyperthermia and showed this was mediated by activation of AMPK and inactivation of mTOR [21]. Metformin is also known to kill and radiosensitize cancer cells, and to preferentially kill breast cancer stem cells, [24] an effect that has also been observed in GBM [23]. One group reported metformin plus TMZ-based chemotherapy as an adjuvant treatment for WHO grade III and IV malignant gliomas, [25] and another group specifically targeted CSCs of GBM using metformin plus sorafenib [23, 26]. Among possible contributors to the superior survival results observed in the combination group in the present study are 1) the combined actions of the cytotoxic effect of TMZ on tumors and the deteriorated cellular energy metabolism environment induced by HL156A, and 2) the additive inhibitory effect of TMZ and HL156A on the invasive properties of TSs, which results in a less aggressive tumor phenotype. Given the heterogeneity of cancer cells, targeting GBM CSCs by evoking general metabolic stress seems a reasonable approach, as it may provide a less favorable environment for metabolically active tumor cells [27, 28]. Although the well-known biguanide, metformin, may have effects similar to those of HL156A, the delivery of metformin to the brain is limited [29]. HL156A overcomes this shortcoming and shows generally high bioavailability. The prominent survival benefit of *in vivo* combination treatment may be attributable to the excellent penetration ability of HL156A. Further studies on this new compound in combination with conventional therapy in the treatment of malignant tumors are warranted.

The cellular mechanism underlying the effect of HL156A on GBM seems distinct in that the expected AMPK activation and consequent inhibition of the mTOR pathway was not consistently observed in this study. It was reported that cellular sensitivity to metformin also depends on the genetic and mutational backgrounds of the different GB cells [23]. Thus, different genetic background of GSC11 TSs and X01 TSs may explain this discrepancy in the response of those specific pathway. Recently, Liu et al. reported that AMPK is activated in glioblastoma, and the anti-proliferative effect of metformin is AMPK independent [16]. The present study also confirmed increased basal AMPK expression in GSC11 and X01 cells, and similarly, found that AMPK activation and consequent mTOR inhibition apparently underlie the inhibitory effect of HL156A and the combination of HL156A and TMZ on GBM TSs. Instead, HL156A, whether alone or combined with TMZ, compromises glucose uptake, as evidenced by the reduced uptake of ^18^F-FDG following drug treatment; this contrasts with activation of AMPK, which usually leads to an increase in glucose uptake [30]. It has been postulated that certain types of glioma stem cells are maintained by an activated glycolytic metabolism [31], and glycolytic glioma cells with active glycogen synthase are further proposed to be sensitive to inhibitors of gluconeogenesis such as metformin [27]. It has been suggested that metformin suppresses gluconeogenesis by inhibiting mitochondrial glycerophosphate dehydrogenase [32]. Metformin also impairs glycolysis by inhibiting hexokinase II [33]. Similarly, the effects of HL156A and the combination of HL156A and TMZ on TSs by can be explained by their inhibitory effects on intracellular glucose metabolism, probably through targeting both mitochondrial and glycolytic pathways. As previous studies revealed CSCs usually showed less OCR compared with their differentiated progenitors [34], the loss of stemness by HL156A, TMZ and combination treatment may affect the reduction in OCR.

Another unique aspect of the current work is that the inhibitory effect of HL156A on the invasive properties of GBM TSs may be related to alterations of EMT-related markers. Several groups have reported that EMT-related markers are upregulated in GBM, and shown that acquisition of mesenchymal traits by cancer cells undergoing EMT is related to the acquisition of a stem cell program [35, 36]. At this point, evidence supporting a role for the EMT pathway in the pathogenesis of GBM expansion and invasion is not robust. Although the process of GBM invasion shares certain similarities with that of immature neuron migration during embryonic development, little is known about the process, and direct evidence for a relation between EMT and invasive properties is still lacking [35]. Nevertheless, several lines of evidence suggest that similar molecular alterations occur with GBM pathophysiology; thus, the effects of inhibiting this pathway need to be studied to identify the role of this pathway in the invasive properties of this fatal disease. An inhibitory effect of metformin on the migration of the U87 glioma cell line has been reported [27]. Similar experiments targeting the EMT pathway using an RNA interference (RNAi) strategy (siRNA or shRNA) may help resolve these unanswered questions.

Even among TMZ-responsive patients, death ultimately ensues owing to relapse of the disease [1]. Several strategies have been proposed for overcoming therapeutic limitation of GBMs, but none has yet proved successful [17–19]. Because of the intrinsic tendency of the tumor to infiltrate into normal brain tissue, complete surgical resection would appear to be an unattainable goal [37]. Thus, appropriate adjuvant therapy for the potential remaining cancer cells, including CSCs, is crucial to overcoming the inevitable fate of the disease. Targeting GBM TSs as a new strategy needs to be extensively evaluated in this context. A caveat to applying this concept is the limited information regarding the identity of GBM-specific stem cells, which makes it a challenge to sort these cells for experimentation. For example, which surface markers define CSCs is a matter of controversy [38]. Furthermore, some properties of GBM can be explained by the clonal evolution model of the disease, which provides another axis of tumor heterogeneity [39]. Heterogeneity defined by the spatial structure of a tumor is better explained by the clonal evolution model. A combined model has also been suggested; thus, more evidence is needed to understand this specific aspect of the disease. Altering general intracellular energy metabolism seems to be a reasonable therapeutic strategy since both models predict that this approach could effectively target neoplastic cells [28]. Accordingly, the use of the newly developed agent, HL156A, combined with conventional TMZ for targeting GBM CSCs, holds promise as a new line of therapy against the disease.

## MATERIALS AND METHODS

### *In vivo* blood brain barrier permeability

ICR mice (Saeronbio, Uiwang, Korea) were used for pharmacokinetic studies. The blood-brain barrier permeability of HL156 and the bioavailability of orally administered drug(30 mg/kg) were assessed by injecting mice intraperitoneally with HL156A (10 mg/kg) and evaluating the concentration of drug in the plasma and brain by liquid chromatography-mass spectrometry.

### GBM TS culture

The GBM TS cell line GSC11, TS13-20 and TS15-88 derived from primary GBM specimens using a previously described method [4, 40] (GSC11 was provided by Frederick F. Lang, M. D. Anderson Cancer Center, Houston, Texas, USA) and the X01 line, derived from a female glioblastoma patient, were used for experiment [41]. For spheres culture, all GBM TSs (GSC11, X01 sphere) were cultured in TS complete media composed of Dulbecco's Modified Eagle Medium/Nutrient Mixture F-12 (DMEM/F-12; Mediatech, Manassas, VA, USA) containing B27 supplements (1×; Invitrogen, San Diego, CA, USA), 20 ng/ml of basic fibroblast growth factor (bFGF; Sigma, St. Louis, MO, USA), 20 ng/ml of epidermal growth factor (EGF; Sigma), and 50 U/ml penicillin/50 mg/ml streptomycin [40, 42, 43].

### Lentiviral vector transduction and expression

GSC11 cells stably expressing green fluorescent protein (GFP-GSC11) and lucerferase(GFP-luc) were generated by growing cells in complete medium and then applying supernatants containing GFP/luciferase-expressing lentivirus. Polybrene (Sigma, Dorset, UK) was added to a final concentration of 8 μg/ml and incubated with cells for 18 hours. After infection, the cells were placed in fresh growth medium and cultured using standard methods. Stable GFP/luciferase-GSC11 cells were selected by culturing with 1 mg/ml puromycin (Life Technologies Korea, Seoul, Korea), and isolated by fluorescence-activated cell sorting (FACS) for use in further experiments.

### Cell viability assay

Effects of HL156A, TMZ, and combined HL156A and TMZ treatment on cell survival was determined using MTS [3-(4,5-dimethylthiazol-2-yl)-5-(3-carboxymethoxyphenyl)-2-(4-sulfophenyl)-2H-tetrazolium, inner salt] assays [44]. Briefly, GBM TS cells (5 × 10^3^) were seeded in 96-well plates, incubated at 37°C for 24 hours, and then treated with HL156A, TMZ, or both for 5 days. MTS reagent (20 μl/well) was added and absorbance was measured at 490 nm after incubating at 37°C for 4 hours. Each experiment was repeated three times in triplicate, and the results were expressed as the percentage of viable cells relative to controls.

### Western blotting

An equal amount of total protein from each sample (20 μg) in Laemmli sample buffer (GeneTex, Irvine, CA, USA) was heated at 100°C for 5 minutes, then resolved by sodium dodecyl sulfate-polyacrylamide gel electrophoresis (SDS-PAGE) on 8% gels and electroblotted onto nitrocellulose membranes (GE Healthcare life-Sciences). Membranes were blocked in Tris-buffered saline containing 0.1% Tween-20 (TBS-T) and 5% non-fat dry milk, and incubated overnight at 4°C with antibodies for AMPK-mammalian target of rapamycin (mTOR) pathway-related proteins, stemness markers and EMT-related markers, and then probed with peroxidase-conjugated goat anti-rabbit IgG (1:2000; Santa Cruz Biotechnology, Inc., Santa Cruz, CA, USA) for 1 hour at room temperature. After repeated washing, membranes were developed using enhanced chemiluminescent (ECL) reagents (Amersham Life Science, Inc., Piscataway, NJ, USA). Band densities were measured using TINA imaging software (Raytest, Straubenhardt, Germany).

### Glioma tumorsphere-formation assay

After producing a single-cell suspension, GBM-TSs (GSC11, TS13-20, TS15-88, U87 sphere and X01) were cultured in 96-well plates in medium consisting of DMEM/F-12 containing 2% 1× B27 supplements, 20 ng/ml of 0.02% bFGF, 20 ng/ml of 0.02% EGF, and 1% antibiotic–antimycotic solution (100×; Gibco, Invitrogen Korea, Seoul, South Korea). The number of sphere-positive wells was counted, and the proportion of sphere-positive wells in the treatment group relative to that in controls was calculated and presented as a percentage. Cells cultured under different conditions for 3 weeks were observed with an inverted phase-contrast microscope (I×71 Inverted Microscope; Olympus, Tokyo, Japan) to determine TS morphology and size, and imaged with a digital camera (DP70 Digital Microscope Camera; Olympus) using DP Controller software (Olympus).

### Three-dimensional invasion assay

GFP-GSC11, GFP-TS13-20, GFP-TS15-88, GFP-U87 sphere and GFP-X01 cells grown as spheroids were cultured in collagen I matrices using polydimethylsiloxane (PDMS)-based microwells (diameter and depth of microwells: 6 mm × 500 μm). Microwells were treated with 1% poly(ethyleneimine) (Sigma-Aldrich, St. Louis, MO, USA) solution for 10 minutes followed by 0.1% glutaraldehyde (Sigma-Aldrich) for 30 minutes, and then washed overnight with phosphate buffered saline (PBS) to make PDMS wells adherent to collagen. Collagen I matrices were prepared from high-concentration rat tail collagen I (BD Bioscience, Le Pont de Claix, France) as recommended by the manufacturer. Briefly, the appropriate amounts of 10x PBS, 1N NaOH, sterile distilled H_2_O, and collagen I were mixed to create gels with the desired final concentration. The solution was mixed well and kept at 4°C before use. GFP-GSC11 spheroids were encapsulated by pipetting 10 μl of collagen I solution (4 mg/ml) into the microwell, placing a single GFP-GSC11 spheroid from the culture plate onto collagen I matrices, and then dropping 10 μl of collagen I solution (4 mg/ml) onto the GFP-GSC11 spheroid. The platform was incubated at 37°C and 5% CO_2_ for 30 minutes. Cell viability was assessed by staining GFP-GSC11 spheroids with 8 μM ethidium homodimer-1 (Invitrogen Korea, Seoul, Korea) for 30 minutes at 37°C before implantation in collagen matrix. After full gelation, culture medium which is consisted of DMEM/F-12 containing 2% 1× B27 supplements, 20 ng/ml of 0.02% bFGF, 20 ng/ml of 0.02% EGF, and 1% antibiotic–antimycotic solution (100×; Gibco) was then added. Cell viability was assessed by staining GFP-GSC11 spheroids with 8 μM ethidium homodimer-1 (Invitrogen Korea, Seoul, Korea) for 30 minutes at 37°C after implantation in collagen matrix. Drug effects were assessed by mixing drugs with medium to the final desired concentration of each drug. The dynamic morphology of GFP-GSC11 spheroids was monitored by obtaining images using an inverted laser-scanning confocal microscope (Nikon Ti-E; Tokyo, Japan). Invasiveness was quantified using the maximal area covered by migrating edges of cells as a parameter, calculated as (invaded area at a certain time/spheroid area at initial time) × 100. Data were analyzed using ImageJ image analysis software (NIH, Bethesda, MD, USA).

### LDH assay

Cytotoxicity was assessed by measuring LDH released into culture media. Cells were plated in 96-well plates at a density of 10 cells per well. After 24 h, cells were treated with 2DG (4 mM), metformin (5 mM or 15 mM), a combination of 2DG (4 mM) and metformin (5 mM) for 3 weeks. The amount of LDH in the medium was determined using a CytoTox 96® Non-Radioactive Cytotoxicity Assay (Promega, Fitchburg, WI, USA) according to the manufacturer's instructions. 10μl of Lysis Solution (10X) per 100μl of medium was added to each well followed by incubation for 45 minutes in a humidified chamber at 37°C, 5% CO2. After centrifuge of the plate at 250 × g for 4 minutes, 50μl aliquots were transferred to a fresh 96-well flat-bottom (enzymatic assay) plate. After adding 50μl of the reconstituted Substrate Mix, the plate was covered with foil to protect it from light, incubated at room temperature for 30 minutes, followed by the addition of 50μl of Stop Solution. Absorbance at 490 nm was determined using a microplate reader.

### ATP assay

For the comparison of ATP levels in TS, the CellTiter-Glo® Luminescent Cell Viability Assay (Promega, Fitchburg, WI, USA) was used. Cells were plated in 96-well plates at a density of 5 × 10^3^ cells per well. After 24 h, they were treated with 4 mM of 2DG, 5 mM of metformin alone, their combination or 15 mM of metformin for 3 days. ATP assay was conducted according to the manufacturer's protocol. A volume of CellTiter-Glo® Reagent equal to the volume of cell culture medium present in each well was added. Cells were incubated at room temperature for 10 minutes, and luminescence was recorded.

### Uptake of ^18^F-FDG

GSC11 cells were plated on 12-well plates at 3 × 10^5^ cells per well, and incubated for 24 hours. The medium was changed to glucose-free DMEM (Gibco) containing approximately 0.5 μCi of ^18^F-fluorodeoxyglucose (^18^F-FDG), and then cells were incubated for 15 minutes. The cells were washed three times with PBS, and 0.1 ml of lysis buffer was added to each well. The lysed cells were then harvested, and the amount of radioactivity in lysates was measured using a Wallac 148 Wizard 3 gamma-counter (PerkinElmer Life and Analytical Science, Shelton, CT, USA) and normalized to protein content.

### Gene expression microarray and gene ontology analysis

Total RNA was extracted from 100 mg of GBM-TSs using a Qiagen miRNA kit according to the manufacturer's protocol. Expression profiles of drug-treated groups and controls were obtained using Illumina HumanHT-12 v4 Expression BeadChips (Illumina, Inc., San Diego, CA, USA). Data were log2 transformed and normalized according to the quantile normalization method using BRB-ArrayTools developed by Dr. Richard Simon and the BRB-ArrayTools Development Team. Genes showing minimal variation across the set of arrays were excluded from the analysis. Genes whose expression differed from the median by at least 1.5-fold in at least 20% of the arrays were retained. Heatmaps were generated and gene ontology analyses were performed using BRB-ArrayTools and GENE-E from broad institute.

### Orthotopic xenograft animal model for the assessment of survival

Male athymic nude mice (4–8 weeks old; Central Lab. Animal Inc., Seoul, Korea) were used for experiments. Mice were housed in micro-isolator cages under sterile conditions and observed for at least 1 week before study initiation to ensure proper health. Lighting, temperature, and humidity were controlled centrally. All experimental procedures were approved by our Institutional Animal Care and Use Committee. Mice were anesthetized with a solution of Zoletil (30 mg/kg; Virbac Korea, Seoul, Korea) and xylazine (10 mg/kg; Bayer Korea, Seoul, Korea) delivered intraperitoneally. GBM TSs (GSC11) were implanted into the right frontal lobe of nude mice using a guide-screw system within the skull. Mice received 5 × 10^5^ cells via a Hamilton syringe (Dongwoo Science Co., Seoul, Korea) inserted to a depth of 4.5 mm. Then, HL156A (30 or 45 mg/kg), TMZ (30 mg/kg), their combination, metformin(500 mg/kg) and metformin and TMZ combination were administrated to mice (n = 5 mice/group). HL156A and metformin was administered orally every other day throughout the duration of the experiment, and TMZ was administrated intraperitoneally for 5 days beginning the day of TS injection. The body weights of mice were checked every other day. If weight decreased by more than 15% compared to the original body weight, mice were euthanized according to the approved protocol. At the end of the experiment, mice were euthanized and their brains were carefully removed, sectioned, mounted on glass slides and stained with hematoxylin and eosin (H&E) for microscopic analysis of gliomagenesis.

### Bioluminescence imaging of animal for the asssement of tumor volume

Bioluminescence acquisition and analysis were performed with the IVIS Imaging System and Living Image V4.2 software. GSC-luc animals were generated as previously described [22] and injected intraperitoneally with d-luciferin (30 mg/mL DPBS, 100μL) at 15 min prior to signal acquisition (5 seconds), which took place under 2.5% isoflurane anesthesia. Grayscale photographic images and bioluminescence color images were superimposed.

### Statistical analysis

Data were analyzed using SPSS for Windows, Version 12.0 (SPSS Inc., Chicago, IL, USA). Student's t-test or Mann–Whitney U test was used for comparison of mean viability and FDG uptake of agent by treated cells. Two-way analysis of variance (ANOVA) was used to detect synergistic effects of combination treatment. Kaplan–Meier survival curves and log-rank statistics were employed for survival analyses. *P*-values < .05 were considered statistically significant.

## SUPPLEMENTARY MATERIALS FIGURES, VIDEOS AND TABLES









## References

[1] Stupp R, Mason WP, van den Bent MJ, Weller M, Fisher B, Taphoorn MJ, Belanger K, Brandes AA, Marosi C, Bogdahn U, Curschmann J, Janzer RC, Ludwin SK (2005). European Organisation for R, Treatment of Cancer Brain T, Radiotherapy G, National Cancer Institute of Canada Clinical Trials G. Radiotherapy plus concomitant and adjuvant temozolomide for glioblastoma. N Engl J Med.

[2] Louis Burt Nabors MD, Network NCC (2014). NCCN Guidelines Version 2.2014 Updates Cental Nervous System Cancers.

[3] Kang SG, Cheong JH, Huh YM, Kim EH, Kim SH, Chang JH (2015). Potential use of glioblastoma tumorsphere: clinical credentialing. Arch Pharm Res.

[4] Sulman E, Aldape K, Colman H (2008). Brain tumor stem cells. Curr Probl Cancer.

[5] Donnenberg VS, Donnenberg AD (2005). Multiple drug resistance in cancer revisited: the cancer stem cell hypothesis. J Clin Pharmacol.

[6] Drewa T, Styczynski J, Szczepanek J (2008). Is the cancer stem cell population “a player” in multi-drug resistance?. Acta Pol Pharm.

[7] Duru N, Candas D, Jiang G, Li JJ (2014). Breast cancer adaptive resistance: HER2 and cancer stem cell repopulation in a heterogeneous tumor society. J Cancer Res Clin Oncol.

[8] Bao S, Wu Q, McLendon RE, Hao Y, Shi Q, Hjelmeland AB, Dewhirst MW, Bigner DD, Rich JN (2006). Glioma stem cells promote radioresistance by preferential activation of the DNA damage response. Nature.

[9] Liu G, Yuan X, Zeng Z, Tunici P, Ng H, Abdulkadir IR, Lu L, Irvin D, Black KL, Yu JS (2006). Analysis of gene expression and chemoresistance of CD133+ cancer stem cells in glioblastoma. Mol Cancer.

[10] Chen K, Huang YH, Chen JL (2013). Understanding and targeting cancer stem cells: therapeutic implications and challenges. Acta Pharmacol Sin.

[11] Cufi S, Vazquez-Martin A, Oliveras-Ferraros C, Martin-Castillo B, Joven J, Menendez JA (2010). Metformin against TGFbeta-induced epithelial-to-mesenchymal transition (EMT): from cancer stem cells to aging-associated fibrosis. Cell Cycle.

[12] Pollak M (2013). Potential applications for biguanides in oncology. J Clin Invest.

[13] Hirsch HA, Iliopoulos D, Tsichlis PN, Struhl K (2009). Metformin selectively targets cancer stem cells, and acts together with chemotherapy to block tumor growth and prolong remission. Cancer Res.

[14] Nies AT, Koepsell H, Winter S, Burk O, Klein K, Kerb R, Zanger UM, Keppler D, Schwab M, Schaeffeler E (2009). Expression of organic cation transporters OCT1 (SLC22A1) and OCT3 (SLC22A3) is affected by genetic factors and cholestasis in human liver. Hepatology.

[15] Ju KD, Kim HJ, Tsogbadrakh B, Lee J, Ryu H, Cho EJ, Hwang YH, Kim K, Yang J, Ahn C, Oh KH (2016). HL156A, a novel AMP-activated protein kinase activator, is protective against peritoneal fibrosis in an in vivo and in vitro model of peritoneal fibrosis. Am J Physiol Renal Physiol.

[16] Liu X, Chhipa RR, Pooya S, Wortman M, Yachyshin S, Chow LM, Kumar A, Zhou X, Sun Y, Quinn B, McPherson C, Warnick RE, Kendler A, Giri S, Poels J, Norga K, Viollet B, Grabowski GA, Dasgupta B (2014). Discrete mechanisms of mTOR and cell cycle regulation by AMPK agonists independent of AMPK. Proc Natl Acad Sci U S A.

[17] Thomas AA, Brennan CW, DeAngelis LM, Omuro AM (2014). Emerging Therapies for Glioblastoma. JAMA Neurol.

[18] Chinot OL, Wick W, Mason W, Henriksson R, Saran F, Nishikawa R, Carpentier AF, Hoang-Xuan K, Kavan P, Cernea D, Brandes AA, Hilton M, Abrey L, Cloughesy T (2014). Bevacizumab plus radiotherapy-temozolomide for newly diagnosed glioblastoma. N Engl J Med.

[19] Gilbert MR, Dignam JJ, Armstrong TS, Wefel JS, Blumenthal DT, Vogelbaum MA, Colman H, Chakravarti A, Pugh S, Won M, Jeraj R, Brown PD, Jaeckle KA (2014). A randomized trial of bevacizumab for newly diagnosed glioblastoma. N Engl J Med.

[20] Stupp R, Hegi ME, Mason WP, van den Bent MJ, Taphoorn MJ, Janzer RC, Ludwin SK, Allgeier A, Fisher B, Belanger K, Hau P, Brandes AA, Gijtenbeek J (2009). European Organisation for R, Treatment of Cancer Brain T, Radiation Oncology G, National Cancer Institute of Canada Clinical Trials G. Effects of radiotherapy with concomitant and adjuvant temozolomide versus radiotherapy alone on survival in glioblastoma in a randomised phase III study: 5-year analysis of the EORTC-NCIC trial. Lancet Oncol.

[21] Lee H, Park HJ, Park CS, Oh ET, Choi BH, Williams B, Lee CK, Song CW (2014). Response of breast cancer cells and cancer stem cells to metformin and hyperthermia alone or combined. PLoS One.

[22] Kim EH, Lee JH, Oh Y, Koh I, Shim JK, Park J, Choi J, Yun M, Jeon JY, Huh YM, Chang JH, Kim SH, Kim KS, Cheong JH, Kim P, Kang SG (2016). Inhibition of glioblastoma tumorspheres by combined treatment with 2-deoxyglucose and metformin. Neuro Oncol.

[23] Yu Z, Zhao G, Xie G, Zhao L, Chen Y, Yu H, Zhang Z, Li C, Li Y (2015). Metformin and temozolomide act synergistically to inhibit growth of glioma cells and glioma stem cells in vitro and in vivo. Oncotarget.

[24] Song CW, Lee H, Dings RP, Williams B, Powers J, Santos TD, Choi BH, Park HJ (2012). Metformin kills and radiosensitizes cancer cells and preferentially kills cancer stem cells. Sci Rep.

[25] Soritau O, Tomuleasa C, Aldea M, Petrushev B, Susman S, Gheban D, Ioani H, Cosis A, Brie I, Irimie A, Kacso G, Florian IS (2011). Metformin plus temozolomide-based chemotherapy as adjuvant treatment for WHO grade III and IV malignant gliomas. J BUON.

[26] Aldea MD, Petrushev B, Soritau O, Tomuleasa CI, Berindan-Neagoe I, Filip AG, Chereches G, Cenariu M, Craciun L, Tatomir C, Florian IS, Crivii CB, Kacso G (2014). Metformin plus sorafenib highly impacts temozolomide resistant glioblastoma stem-like cells. J BUON.

[27] Beckner ME, Gobbel GT, Abounader R, Burovic F, Agostino NR, Laterra J, Pollack IF (2005). Glycolytic glioma cells with active glycogen synthase are sensitive to PTEN and inhibitors of PI3K and gluconeogenesis. Lab Invest.

[28] Kim SY (2015). Cancer metabolism: targeting cancer universality. Arch Pharm Res.

[29] Menendez JA, Quirantes-Pine R, Rodriguez-Gallego E, Cufi S, Corominas-Faja B, Cuyas E, Bosch-Barrera J, Martin-Castillo B, Segura-Carretero A, Joven J (2014). Oncobiguanides: Paracelsus' law and nonconventional routes for administering diabetobiguanides for cancer treatment. Oncotarget.

[30] O'Neill HM (2013). AMPK and Exercise: Glucose Uptake and Insulin Sensitivity. Diabetes Metab J.

[31] Mao P, Joshi K, Li J, Kim SH, Li P, Santana-Santos L, Luthra S, Chandran UR, Benos PV, Smith L, Wang M, Hu B, Cheng SY, Sobol RW, Nakano I (2013). Mesenchymal glioma stem cells are maintained by activated glycolytic metabolism involving aldehyde dehydrogenase 1A3. Proc Natl Acad Sci U S A.

[32] Madiraju AK, Erion DM, Rahimi Y, Zhang XM, Braddock DT, Albright RA, Prigaro BJ, Wood JL, Bhanot S, MacDonald MJ, Jurczak MJ, Camporez JP, Lee HY, Cline GW, Samuel VT, Kibbey RG, Shulman GI (2014). Metformin suppresses gluconeogenesis by inhibiting mitochondrial glycerophosphate dehydrogenase. Nature.

[33] Salani B, Del Rio A, Marini C, Sambuceti G, Cordera R, Maggi D (2014). Metformin, cancer and glucose metabolism. Endocr Relat Cancer.

[34] Pattappa G, Heywood HK, de Bruijn JD, Lee DA (2011). The metabolism of human mesenchymal stem cells during proliferation and differentiation. J Cell Physiol.

[35] Ortensi B, Setti M, Osti D, Pelicci G (2013). Cancer stem cell contribution to glioblastoma invasiveness. Stem Cell Res Ther.

[36] Kalluri R, Weinberg RA (2009). The basics of epithelial-mesenchymal transition. J Clin Invest.

[37] Tanahashi K, Natsume A, Ohka F, Momota H, Kato A, Motomura K, Watabe N, Muraishi S, Nakahara H, Saito Y, Takeuchi I, Wakabayashi T (2014). Assessment of tumor cells in a mouse model of diffuse infiltrative glioma by Raman spectroscopy. Biomed Res Int.

[38] Jordan CT (2009). Cancer stem cells: controversial or just misunderstood?. Cell Stem Cell.

[39] Shlush LI, Hershkovitz D (2015). Clonal evolution models of tumor heterogeneity. Am Soc Clin Oncol Educ Book.

[40] Kong BH, Park NR, Shim JK, Kim BK, Shin HJ, Lee JH, Huh YM, Lee SJ, Kim SH, Kim EH, Park EK, Chang JH, Kim DS, Kim SH, Hong YK, Kang SG, Lang FF (2013). Isolation of glioma cancer stem cells in relation to histological grades in glioma specimens. Childs Nerv Syst.

[41] Soeda A, Park M, Lee D, Mintz A, Androutsellis-Theotokis A, McKay RD, Engh J, Iwama T, Kunisada T, Kassam AB, Pollack IF, Park DM (2009). Hypoxia promotes expansion of the CD133-positive glioma stem cells through activation of HIF-1alpha. Oncogene.

[42] Singh SK, Hawkins C, Clarke ID, Squire JA, Bayani J, Hide T, Henkelman RM, Cusimano MD, Dirks PB (2004). Identification of human brain tumour initiating cells. Nature.

[43] Kwak J, Shin HJ, Kim SH, Shim JK, Lee JH, Huh YM, Kim EH, Park EK, Chang JH, Kim SH, Hong YK, Kim DS, Lee SJ, Kang SG (2013). Isolation of tumor spheres and mesenchymal stem-like cells from a single primitive neuroectodermal tumor specimen. Childs Nerv Syst.

[44] Mosmann T (1983). Rapid colorimetric assay for cellular growth and survival: application to proliferation and cytotoxicity assays. Journal of immunological methods.

