# Supplementary material for: Inhibiting stemness and invasive properties of glioblastoma tumorsphere by combined treatment with temozolomide and a newly designed biguanide (HL156A)
Source: Oncotarget. 2016 Aug 25;7(40):65643–59. doi: 10.18632/oncotarget.11595 (PMC5323181; doi:10.18632/oncotarget.11595)
Supplement: Supplementary file 1 [file oncotarget-07-65643-s001.pdf]

Inhibiting stemness and invasive properties of glioblastoma tumorsphere by combined treatment with temozolomide and a newly designed biguanide (HL156A)

SUPPLEMENTARY FIGURES, VIDEOS AND TABLES

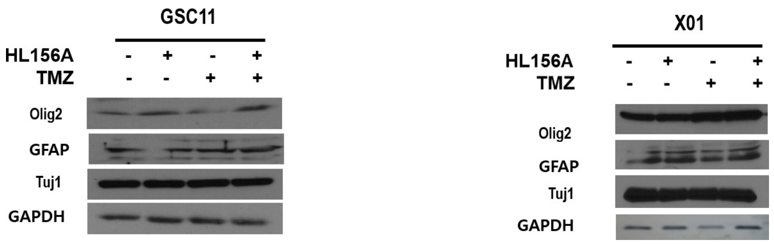

Supplementary Figure S1: Expression of differentiation markers in GSC11 and X01 sphere. The treatment of HL156A, TMZ and combination did not alter the expression of differentiation markers.

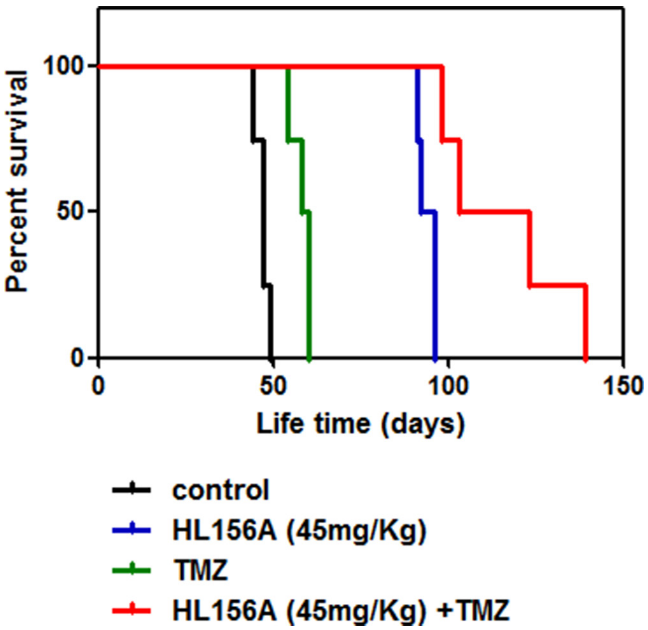

Supplementary Figure S2: Kaplan-Meier curve of Mice treated with high dose of HL156A(45 mg/kg), TMZ and combination. Significant survival benefit was observed in HL156A and combination treatment group.

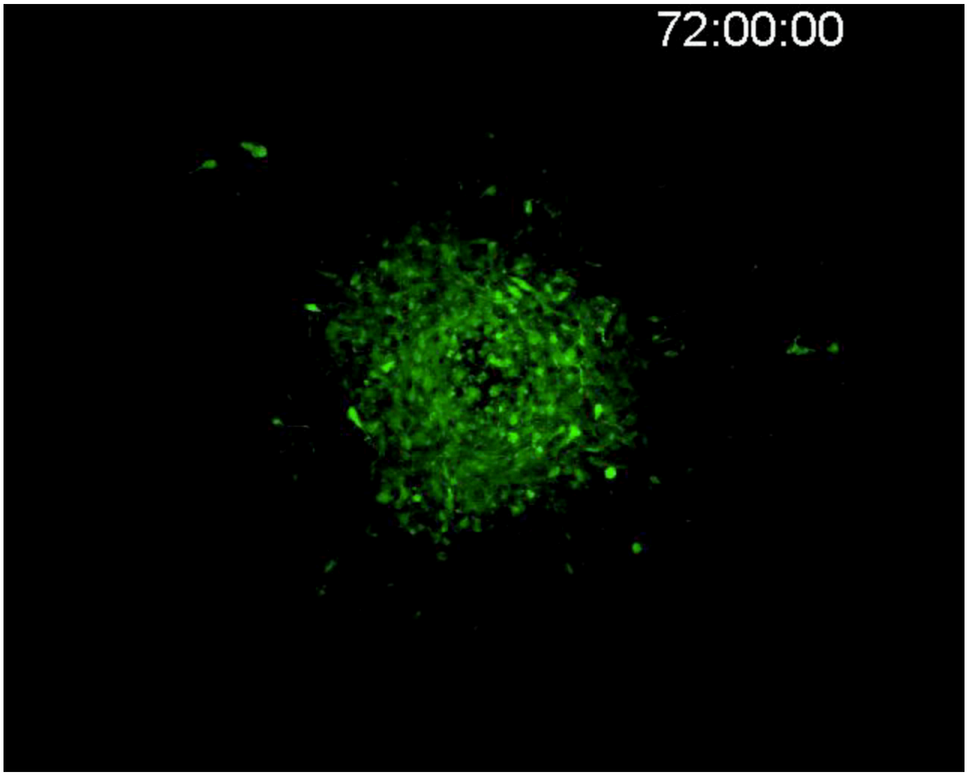

Supplementary Video S1: Invasion of GSC11 TS in spheroid into 3D collagen-based matrix without treatment of drugs. GSC11 TS infiltrated dispersedly into collagen-based matrix (confocal GFP image, 65hr movie, 15min interval, 25fpsm, scale bar:100µm).

See Supplementary Video S1

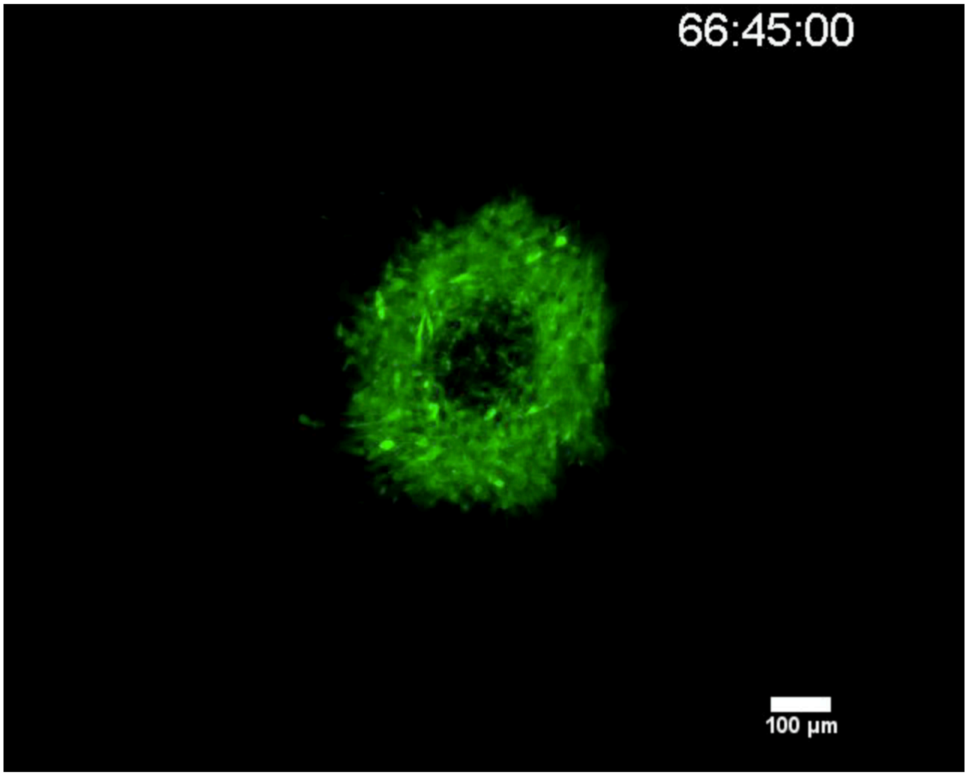

Supplementary Video S2: Invasion of GSC11 TS in spheroid into 3D collagen-based matrix with treatment of HL156 and TMZ. Infiltration of GSC11 TS into 3D collagen-based matrix was inhibited by the treatment of the combination of HL156(15 µM) and TMZ (500 µM) (confocal GFP image, 65hr movie, 15min interval, 25fpsm, scale bar:100µm).

See Supplementary Video S2

Supplementary Table S1: Pharmacokinetics of HL156A. Pharmacokinetic parameters of HL156A in mice were given

**Route**	**IV**		**PO**	
**Concentration (mg/kg)**	**10**	**3**	**10**	**30**
AUC0-t(ng.hr/ml)	1312.1	26.6	472.6	2859.2
AUC0-∞(ng.hr/ml)	1356	nc	504.6	2966
Cmax (ng/ml)	-	24.1	265	961
Tmax (h)	-	0.75	0.42	0.92
Cl (ml/min/kg)	124.9	-	-	-
Vss (L/kg)	11.9	-	-	-
Vz (L/kg)	18.5	-	-	-
T1/2 (h)	1.7	nc	1.3	1.7

nc : Not congnizable

Supplementary Table S2: Results of GO analysis. Differentially expressed gene sets between TMZ and combination treatment. Expression of several gene sets related cellular adhesion and migration was significantly different between two groups.

See Supplementary File 1
