# Supplementary material for: Inhibiting stemness and invasive properties of glioblastoma tumorsphere by combined treatment with temozolomide and a newly designed biguanide (HL156A)
Source: Oncotarget. 2016 Aug 25;7(40):65643–59. doi: 10.18632/oncotarget.11595 (PMC5323181; doi:10.18632/oncotarget.11595)
Supplement: Supplementary file 2 [file oncotarget-07-65643-s002.doc]

**Supplement Table 2. Results of GO analysis**

|  | GO category | GO ontology | GO term | Number of genes | LS permutation p-value | KS permutation p-value | Efron-Tibshirani's GSA test p-value |
| --- | --- | --- | --- | --- | --- | --- | --- |
| 1 | GO:0007155 | BP | **cell adhesion** | 88 | 0.00003 | 0.24119 | 0.04 (+) |
| 2 | GO:0022610 | BP | **biological adhesion** | 88 | 0.00003 | 0.24119 | 0.04 (+) |
| 3 | GO:0048699 | BP | generation of neurons | 96 | 0.00008 | 0.00622 | < 0.005 (-) |
| 4 | GO:0050839 | MF | **cell adhesion molecule binding** | 22 | 0.00023 | 0.05207 | 0.11 (+) |
| 5 | GO:0001558 | BP | regulation of cell growth | 29 | 0.00023 | 0.00411 | < 0.005 (+) |
| 6 | GO:0045595 | BP | regulation of cell differentiation | 92 | 0.00029 | 0.0322 | 0.095 (+) |
| 7 | GO:0048870 | BP | **cell motility** | 93 | 0.00035 | 0.15429 | < 0.005 (+) |
| 8 | GO:0051674 | BP | localization of cell | 93 | 0.00035 | 0.15429 | < 0.005 (+) |
| 9 | GO:0016477 | BP | **cell migration** | 92 | 0.00041 | 0.16148 | < 0.005 (+) |
| 10 | GO:0034330 | BP | cell junction organization | 12 | 0.00044 | 0.00094 | < 0.005 (-) |
| 11 | GO:0030182 | BP | neuron differentiation | 86 | 0.00046 | 0.01401 | < 0.005 (-) |
| 12 | GO:0000902 | BP | cell morphogenesis | 82 | 0.00047 | 0.032 | 0.205 (+) |
| 13 | GO:0016337 | BP | **single organismal cell-cell adhesion** | 28 | 0.0005 | 0.24627 | < 0.005 (+) |
| 14 | GO:0009611 | BP | response to wounding | 68 | 0.0005 | 0.07895 | 0.07 (+) |
| 15 | GO:0016192 | BP | vesicle-mediated transport | 73 | 0.00061 | 0.00701 | 0.335 (+) |
| 16 | GO:0048666 | BP | neuron development | 67 | 0.00069 | 0.06203 | < 0.005 (-) |
| 17 | GO:1903035 | BP | negative regulation of response to wounding | 9 | 0.00072 | 0.00408 | < 0.005 (+) |
| 18 | GO:0005178 | MF | **integrin binding** | 13 | 0.00072 | 0.01091 | < 0.005 (+) |
| 19 | GO:0006897 | BP | endocytosis | 34 | 0.00074 | 0.00291 | 0.435 (+) |
| 20 | GO:0030155 | BP | **regulation of cell adhesion** | 34 | 0.00077 | 0.40607 | < 0.005 (+) |
| 21 | GO:0045216 | BP | cell-cell junction organization | 11 | 0.00077 | 0.00179 | < 0.005 (-) |
| 22 | GO:0034097 | BP | response to cytokine | 55 | 0.00078 | 0.10488 | 0.145 (+) |
| 23 | GO:0007160 | BP | cell-matrix adhesion | 12 | 0.0008 | 0.01404 | < 0.005 (-) |
| 24 | GO:0031348 | BP | negative regulation of defense response | 10 | 0.00082 | 0.00278 | < 0.005 (+) |
| 25 | GO:0098602 | BP | **single organism cell adhesion** | 32 | 0.00096 | 0.42385 | < 0.005 (+) |
| 26 | GO:0016049 | BP | cell growth | 38 | 0.00099 | 0.11886 | < 0.005 (+) |
| 27 | GO:0030030 | BP | cell projection organization | 74 | 0.001 | 0.06159 | 0.14 (-) |
| 28 | GO:0032102 | BP | negative regulation of response to external stimulus | 14 | 0.00101 | 0.00215 | 0.12 (+) |
| 29 | GO:0032989 | BP | cellular component morphogenesis | 86 | 0.00115 | 0.08409 | 0.245 (+) |
| 30 | GO:0034329 | BP | cell junction assembly | 10 | 0.00117 | 0.00268 | < 0.005 (-) |
| 31 | GO:0050767 | BP | regulation of neurogenesis | 41 | 0.00133 | 0.08463 | 0.21 (+) |
| 32 | GO:0051093 | BP | negative regulation of developmental process | 51 | 0.00159 | 0.04778 | < 0.005 (+) |
| 33 | GO:0023056 | BP | positive regulation of signaling | 76 | 0.00169 | 0.09863 | < 0.005 (+) |
| 34 | GO:0010647 | BP | positive regulation of cell communication | 77 | 0.00189 | 0.0942 | < 0.005 (+) |
| 35 | GO:0031175 | BP | neuron projection development | 62 | 0.00192 | 0.08327 | 0.08 (+) |
| 36 | GO:0001952 | BP | **regulation of cell-matrix adhesion** | 5 | 0.00197 | 0.001 | < 0.005 (-) |
| 37 | GO:0031589 | BP | **cell-substrate adhesion** | 19 | 0.00206 | 0.2878 | 0.03 (-) |
| 38 | GO:0043405 | BP | regulation of MAP kinase activity | 18 | 0.00226 | 0.00123 | < 0.005 (+) |
| 39 | GO:0050728 | BP | negative regulation of inflammatory response | 7 | 0.00245 | 0.01371 | < 0.005 (+) |
| 40 | GO:2001237 | BP | negative regulation of extrinsic apoptotic signaling pathway | 9 | 0.00251 | 0.01567 | < 0.005 (+) |
| 41 | GO:0000904 | BP | cell morphogenesis involved in differentiation | 64 | 0.00253 | 0.07539 | 0.08 (+) |
| 42 | GO:0043588 | BP | skin development | 24 | 0.0026 | 0.06641 | 0.25 (+) |
| 43 | GO:0007264 | BP | small GTPase mediated signal transduction | 23 | 0.00263 | 0.00184 | 0.21 (-) |
| 44 | GO:0009897 | CC | external side of plasma membrane | 17 | 0.00271 | 0.49503 | 0.055 (+) |
| 45 | GO:0051216 | BP | cartilage development | 13 | 0.00324 | 0.01037 | < 0.005 (+) |
| 46 | GO:0045664 | BP | regulation of neuron differentiation | 33 | 0.00344 | 0.18438 | 0.21 (+) |
| 47 | GO:0007266 | BP | Rho protein signal transduction | 7 | 0.00345 | 0.00382 | < 0.005 (-) |
| 48 | GO:0043406 | BP | positive regulation of MAP kinase activity | 11 | 0.00348 | 0.01644 | < 0.005 (-) |
| 49 | GO:0051960 | BP | regulation of nervous system development | 44 | 0.00351 | 0.26241 | < 0.005 (+) |
| 50 | GO:0061448 | BP | connective tissue development | 17 | 0.00353 | 0.00213 | < 0.005 (+) |
| 51 | GO:0033559 | BP | unsaturated fatty acid metabolic process | 6 | 0.00368 | 0.02054 | < 0.005 (+) |
| 52 | GO:1903034 | BP | regulation of response to wounding | 31 | 0.00371 | 0.48388 | 0.145 (+) |
| 53 | GO:0006909 | BP | phagocytosis | 11 | 0.00395 | 0.00158 | 0.34 (+) |
| 54 | GO:0048864 | BP | stem cell development | 16 | 0.00416 | 0.00813 | < 0.005 (+) |
| 55 | GO:0061024 | BP | membrane organization | 44 | 0.00425 | 0.0609 | 0.035 (+) |
| 56 | GO:0006954 | BP | inflammatory response | 34 | 0.00427 | 0.23222 | 0.145 (+) |
| 57 | GO:0030054 | CC | cell junction | 80 | 0.00438 | 0.06943 | 0.175 (-) |
| 58 | GO:0030098 | BP | lymphocyte differentiation | 13 | 0.00448 | 0.00078 | 0.4 (+) |
| 59 | GO:0048771 | BP | tissue remodeling | 18 | 0.00453 | 0.001 | 0.15 (-) |
| 60 | GO:0022407 | BP | **regulation of cell-cell adhesion** | 13 | 0.00458 | 0.0547 | < 0.005 (+) |
| 61 | GO:0032990 | BP | cell part morphogenesis | 59 | 0.00479 | 0.04231 | 0.14 (-) |
